# The bacteriology of bronchiectasis patients and relation to disease severity

**DOI:** 10.1186/s12890-026-04378-w

**Published:** 2026-06-17

**Authors:** Enas Sayed Farhat, Afnan Mahmoud Abd el Halim, Assem Fouad Elessawy, Radwa Ahmed Elhefny, Mona Ibrahim Ahmed, Amany Mahmoud Ahmed, Samar Ahmed Fouad

**Affiliations:** Faculty of Medicine, Fayoum University, Fayoum, Egypt

**Keywords:** Bronchiectasis, Fayoum, Microbiome

## Abstract

**Background:**

Bronchiectasis is a progressive pulmonary disease with repeated cough, expectoration and frequent respiratory infections. Every patient should have sample collected for routine bacteriological culture. Determining the disease’s severity can help with therapy and follow-up choices.

**Aim of the study:**

To detect the bacteriology of bronchiectasis patients and relation to disease severity.

**Results:**

60 patients with bronchiectasis exacerbation were investigated at chest department of Fayoum University Hospital. Broncho alveolar lavage for culture and sensitivity was done. Disease severity was assessed by cough score, mMRC dyspnea score, oxygen saturation, no of lobes affected in CT chest and modified rieff score, Spirometry and classification of severity by FEV1, and finally FACED and BSI scores were calculated. Isolation of H.Influenza represents 40%, Pseudomonas represents 26.7%, Klebsiella represents 20%, Staph aureus represents 10% and Pseudomonas& Klebsiella represent 3.3%. There was a statistically significant lower mean of oxygen saturation in cases infected with both pseudomonas and Klebsiella. There was a statistically significant high percentage of mild Modified Reiff score among cases infected with H. influenza, moderate degree among cases infected with Klebsiella, but severe degree among cases infected with pseudomonas.

**Conclusion:**

H. influenzae consider as a major pathogen isolated by BAL culture in patients with bronchiectasis exacerbation, followed by P. aeruginosa, then klebsiella then S. aureus. Cases infected with P. aeruginosa and klebsiella have the worst oxygen saturation. The highest modified Rieff score was in P. aeruginosa than other isolated organisms.

## Introduction

The respiratory condition known as bronchiectasis is progressive and diverse. Recurrent respiratory infections, sputum production, and coughing are its hallmarks [1].

By encouraging airway damage and escalating inflammation, infectious agents cause the pathophysiology of bronchiectasis. Additionally, by eluding immune responses of host, microorganisms can remain in the airways and cause resistance to anti-microbial treatment [2].

Instead of using “bronchial colonization,” it is better using “pathogenic colonization,” which is represented as “bronchial infection.” This form of “passive pathogenesis” is brought on by microorganisms growing on the respiratory mucosal surface without compromising the nearby tissues producing a localized inflammatory response [3].

Pseudomonas aeruginosa, Haemophilus influenzae, Streptococcus pneumoniae, Moraxella catarrhalis and Staph aureus are the bacteria that are most commonly identified from the airway secretions of patients who have bronchiectasis [4]. Methicillin-resistant S. aureus (MRSA) in bronchiectasis has not been extensively studied, despite the possibility that its prevalence is increasing [4].

Every patient with bronchiectasis should have a sputum sample taken for routine mycobacterial and bacteriological culture. It is regarded as a straightforward, non-invasive, and affordable test [5].

The disease’s severity can be categorized to help with patient planning, treatment, and follow-up decisions. The FACED and BSI (Bronchiectasis Severity Index) scores are often used severity scores [6].

Additionally, a number of scoring systems, like the modified Reiff score, which characterizes radiological extent and severity, provide a dependable means of tracking the state and course of the disease as measured by chest high-resolution computed tomography (HRCT) [7]. In earlier publications, FEV1 was used to indicate the degree of airway obstruction and the severity of the condition [8].

### The aim of the study

to detect the bacteriology of patients with bronchiectasis and relation to severity of disease.

#### Patients and methods

This observational study was performed in chest department of Fayoum University hospital and included sixty patients admitted at chest department, Fayoum university hospital by exacerbation of bronchiectasis which defined based on the presence of sudden worsening of symptoms including dyspnea, increased sputum volume, or increased sputum purulence with fever, patient became hypoxic or worsening of hypoxia.

All patients gave their informed consent for inclusion in this study.

- Inclusion Criteria:
- Confirmed bronchiectasis diagnosis (CT scan).
- BAL samples collected for bacteriology.
- Exclusion Criteria:
- Antibiotic use in past 2 weeks.
- Cystic fibrosis.
- Active tuberculosis.
- Immunocompromised states.

Every patient included in the study was submitted to:

- 1: Complete history especially history of comorbidities: hypertension, diabetes mellitus, COPD and collagen vascular diseases.
- 2: Complete clinical examination: General and chest examination
- 3: Laboratory evaluation: Oxygen saturation and Laboratory investigation: complete blood count (CBC), coagulation profile, ESR, CRP, liver and kidney function tests.

4: Broncho – alveolar lavage for culture and sensitivity:

- BAL performed with 100-150 ml sterile saline instilled in 3rd-4th generation bronchi.
- Sample collected in sterile containers.
- Bronchial lavage samples were processed using conventional microbiological techniques. Specimens were cultured on blood agar, chocolate agar, and MacConkey agar plates. Chocolate agar was used for the isolation of Haemophilus influenza, while MacConkey agar supported the growth of Gram-negative organisms such as Pseudomonas aeruginosa and Klebsiella pneumonia. All culture plates were incubated at 37 °C for 18–24 h under aerobic conditions. Chocolate agar plates were incubated in a 5% CO₂ atmosphere to facilitate the growth of Haemophilus influenza. Significant bacterial growth was defined as ≥ 10⁴ colony-forming units (CFU)/mL. (CLSI., 2025)

Bacterial growth was evaluated after 18–24 h of incubation. Significant bacterial growth was defined based colony count more than 104 cfu/ ml on the presence of pure or predominant growth of a potential pathogen, consistent colony morphology, and correlation with clinical findings. Mixed growth of normal flora without a predominant organism was considered non-significant.

Preliminary identification of bacterial isolates was performed using Gram staining and colony morphology. Further identification was carried out using standard biochemical tests. For Gram-negative isolates, biochemical identification included triple sugar iron (TSI) agar, lysine iron agar (LIA), and citrate utilization tests, in addition to other standard biochemical reactions used for the identification of Gram-negative bacteria.

For Gram-positive cocci, particularly Staphylococcus species, biochemical identification included catalase testing followed by coagulase, mannitol fermentation on mannitol salt agar, and DNase tests (CLSI., 2025).

Final confirmation of bacterial identification was performed using an automated identification system, VITEK^®^ 2 Compact automated identification system (bioMérieux, Marcy l’Etoile, France).

Antimicrobial susceptibility testing was performed and interpreted according to the guidelines of the Clinical and Laboratory Standards Institute (CLSI., 2025).

5: Radiology: high resolution CT chest without contrast using 160 MSCT Toshiba Aquilion Prime Machine, and the bronchiectasis severity was graded by using modified Reiff score.

The degree and severity are indicated using a grading system known as the Modified Reiff score. It is used due to its ease of use and capacity to assess the dilatation degree as well as the number of implicated lobes. This score depends on the diameter of the vessel and the adjacent bronchus. In each of the six lung lobes (lingula was regarded as a separate lobe), there are zero points (none), one point (cylindrical), two points (varicose), and three points (cystic). Scores vary from 0 to 18, with mild (1–6), moderate (7–12), and severe (13–18) scores being the three categories [9]. 

6: Lung function assessment of FEV1 by spirometry (Spirobank II).

7: Assessment of FACED score, bronchiectasis severity index, cough score and mMRC dyspnea scale shown in (Tables 1, 2 and 3).

**Table 1:** Bronchiectasis severity index and FACED scores [10]

	BSI		FACED score	
FEV1 predicted	>80	0 point	> 50	0 point
	50–80	1point	<50	2 points
	30–49	2 point		
	< 30	3 points		
Age	< 50 years	0 point	<70 years	0 point
	50–69 years	2 points	>70 years	2 points
	70–79 years	4 points		
	> 80 years	6 points		
bacterial colonization (chronic)	p.aeruginosa	3 point	No	0 point
	others	1 point	Yes, p. aeruginosa	1 point
	none	0 point		
Number of lobes affected	< 2	0 points	<2	0 point
	> 2 or cystic BE	1 point	>2	1 point
mMRC dyspnea score	< 3	0 point	<2	0 point
	4	2 points	>2	1 point
	5	3 points		
Body mass index	< 18.5	2 points		
	> 18.5	0 point		
Admissions to hospital in last 2 years	Yes	5 points		
	No	0 point		
Frequency of exacerbations in the previous 1 year	< 3	0 point		
	> 3	2 points		

**Table 2:** Cough score

Score	Day time	Night time
0	No cough	No cough
1	Mild cough in the day time	Mild cough prior to sleep or cough at the night
2	Frequent cough with mild daily Life affection	Mild Cough with night sleep affection
3	Frequent cough with severe daily Life affection	Severe Cough with night sleep affection

**Table 3:** The modified Medical Research Council (mMRC) score [11]. 8: All patients received routine treatment according to ERS bronchiectasis guidelines [12]

Grade	
0	No dyspnea except on severe exercise
1	I get breathlessness on walking up a slight hill or hurrying on the level
2	I walk less than people of the same age on level ground or I stop to catch breath with walking at my own place on the level due to breathlessness
3	Stops for breath with few minutes on the level or with walking about 100 m
4	I am too breathless at leaving house, or I am breathless with dressing or undressing

### Statistical analysis of data

Data collected and coded to facilitate data manipulation and double entered into Microsoft Access and data analysis performed using the Statistical Package of Social Science (SPSS) software version 22 in windows 7 (SPSS Inc., Chicago, IL, USA). Simple descriptive analysis in the form of numbers and percentages of qualitative data, and arithmetic means as central tendency measurement, standard deviations as a measure of dispersion of quantitative parametric data. ANOVA test and kruskal Wallis test used to compare more than two dependent quantitative data, with post Hoc test between pairs. Chi square test used for qualitative data. The *P*-value < 0.05 was considered as statistical significant.

## Results

The table illustrated that the mean age among study group was (45.9 ± 16.1) years old and mean BMI was (24.5 ± 5.9) with 40% were males versus 60% were females. 23.3% were smoker, 10% had diabetes mellitus, and same for hypertension. 21.7% of them were overweight, and 25% were obese. (Table 4).

**Table 4:** Description of demographic characteristics among study group

Variables	Description (*n* = 60)	
	Mean ± SD	Median (Range )
Age (years)	45.9 ± 16.1	46(13–80)
BMI (kg/m2)	24.5 ± 5.9	23.5(15–37)
Sex	**No**	**%**
Male	24	40%
Female	36	60%
Smoking		
Yes	14	23.3%
No	46	76.7%
Comorbidities		
No	28	46.7%
Diabetes mellitus	6	10%
Hypertension	6	10%
Rheumatoid arthritis	1	1.7%
COPD	3	5%
DM& HTN	8	13.3%
DM&HTN&COPD	2	3.3%
DM&COPD	2	3.3%
HTN&RA	1	1.7%
HTN&COPD	3	5%
BMI grading		
Underweight	9	15%
Normal	23	38.3%
Overweight	13	21.7%
Obese	15	25%

Here, the description of clinical data. 40% of cases had mild hypoxia and 16.7% had moderate hypoxia. Regarding degree of obstruction by spirometry: 6.7% had mild FEV1, 30% had moderate FEV1, 35% had severe FEVI and 28.3% had very severe. 95% had mild FACED score, 1.7% with moderate score and 3.3% with severe score.95% had mild BSI score and 5% had moderate score. The main value of Cough score at morning was (2.28 ± 0.61), and (2.62 ± 0.58) at night, mean mMRC dyspnea score was (3.4 ± 0.62) (Table 5).

**Table 5:** Description of clinical data among study group

Variables	Description (*n* = 60)	
	Mean ± SD	Median (Range)
Oxygen saturation	93.02 ± 4.2	94(77–99)
FEV1	44.3 ± 18.3	41.5(17–97)
FACED score	0.22 ± 0.97	0(0–5)
BSI score	0.65 ± 2.9	0(0–16)
O2 saturation severity	**No**	**%**
Normal	26	43.3%
Mild hypoxia	24	40%
Moderate hypoxia	10	16.7%
FEV1 Severity	**No**	**%**
Mild	4	6.7%
Moderate	18	30%
Sever	21	35%
Very sever	17	28.3%
FACED score severity	**No**	**%**
Mild	57	95%
Moderate	1	1.7%
Sever	2	3.3%
BSI score severity	**No**	**%**
Mild	57	95%
Moderate	3	5%
Clinical symptoms	**Mean ± SD**	**Median (Range )**
Cough score at morning	2.28 ± 0.61	2(1–3)
Cough score at night	2.62 ± 0.58	3(1–3)
mMRC dyspnea score	3.4 ± 0.62	3(2–4)

The table illustrated that the 61.7% of cases show secretion in bronchoscope, 56.7% of them show moderate level of Modified Reiff score severity with a mean of (9.1 ± 3.5), and mean CT lobes of (3.15 ± 1.05). (Table 6).

**Table 6:** Description of Bronchoscopy, and radiology findings among study group

Variables	Description (*n* = 60)	
	No	%
Bronchoscopy findings		
No	2	3.3%
Mucus plug	17	28.3%
Secretion	37	61.7%
Hemoptysis	3	5%
Secretion & Hemoptysis	1	1.7%
Radiological findings	**Mean ± SD**	**Median (Range )**
Modified Reiff score	9.1 ± 3.5	8(3–18)
Number of CT lobes	3.15 ± 1.05	3(1–5)
Modified Reiff score severity	**No**	**%**
Mild	17	28.3%
Moderate	34	56.7%
Sever	9	15%

**Fig. 1:**
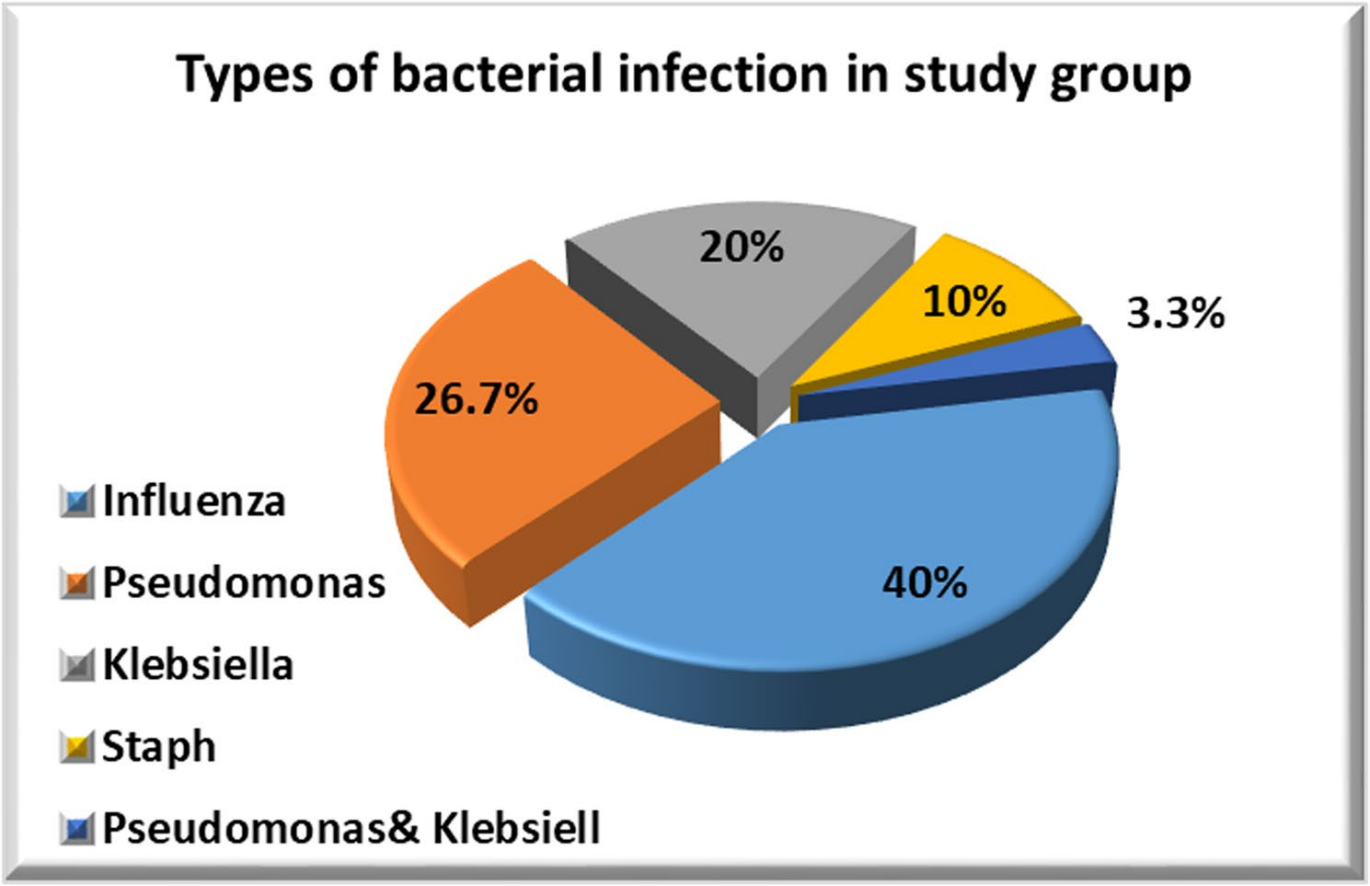
Bacteriology among study group

The mean number of exacerbation was (2.9 ± 0.75) and mean number of hospitalization times last 2 years was (1.3 ± 0.73) with 58.3% of cases show bacterial growth higher percentage was for pseudomonas 40% for H.influenza infection, followed by 26.7% for infection with Pseudomonas. (Table 7) (Fig. 1).

**Table 7:** Frequency of different outcomes among study group

Variables (*n* = 60)	Frequency	
	Mean ± SD	Median (Range)
Number of Exacerbation in last year	2.9 ± 0.75	3(1–5)
Number of hospital admission in last 2 years	1.3 ± 0.73	1(0–3)
Bacteriology	**No**	**%**
H.Influenza	24	40%
Pseudomonas	16	26.7%
Klebsiella	12	20%
Staph	6	10%
Pseudomonas& Klebsiella	2	3.3%

The table illustrated that there was a statistical significant lower mean of oxygen saturation level among cases infected with both pseudomonas and Klebsiella with p-value 0.04. On the other hand, there was no statistical significant difference with p-value > 0.05 as regards other disease severity variables. (Table 8).

**Table 8:** Comparisons of types of growth in different disease severity degrees among cases

Variables	Influenza	pseudomonas	Klebsiella	Staph	Mixed	*P*-value
	No.(%)	No.(%)	No.(%)	No.(%)	No.(%)	
O2 saturation severity						
Normal	14(58.3%)	6(37.5%)	5(41.7%)	1(16.7%)	----	0.38
Mild hypoxia	8(33.3%)	6(37.5%)	6(50%)	3(50%)	1(50%)	
Moderate hypoxia	2(8.3%)	4(25%)	1(8.3%)	2(33.3%)	1(50%)	
FEV1 Severity						
Mild	3(12.5%)	1(6.3%)	----	----	----	0.44
Moderate	9(37.5%)	5(31.3%)	1(8.3%)	2(33.3%)	1(50%)	
Sever	8(33.3%)	7(43.8%)	5(41.7%)	1(16.7%)	----	
Very sever	4(16.7%)	3(18.8%)	6(50%)	3(50%)	1(50%)	
FACED score severity						
Mild	23(95.8%)	15(93.8%)	12(100%)	5(83.3%)	2(100%)	0.59
Moderate	1(4.2%)	----	----	----	----	
Sever	----	1(6.3%)	----	1(16.7%)	----	
BSI score severity						
Mild	23(95.8%)	15(93.8%)	12(100%)	5(83.3%)	2(100%)	0.63
Moderate	1(4.2%)	1(6.3%)	----	1(16.7%)	----	

	Mean ± SD	Mean ± SD	Mean ± SD	Mean ± SD	Mean ± SD	
O2 saturation	94.8 ± 2.8	91.7 ± 5.7	92.8 ± 2.8	91 ± 3.8	89 ± 7.1	0.04*
FEV1	51.6 ± 20.8	43.9 ± 16.2	34.7 ± 10.4	35.5 ± 15.8	42.5 ± 23.3	0.06
FACED score	0.13 ± 0.61	0.31 ± 1.3	0 ± 0	0.83 ± 2.04	0 ± 0	0.49
BSI score	0.4 ± 2.6	0.63 ± 2.5	0 ± 0	2.7 ± 6.5	0 ± 0	0.47
Morning Cough score	2.2 ± 0.65	2.3 ± 0.68	2.3 ± 0.49	2.5 ± 0.54	2.5 ± 0.71	0.83
Night Cough score	2.5 ± 0.65	2.6 ± 0.63	2.9 ± 0.29	2.7 ± 0.52	2.5 ± 0.71	0.37
mMRC dyspnea score	3.3 ± 0.53	3.3 ± 0.79	3.8 ± 0.45	3.5 ± 0.55	3.5 ± 0.71	0.21
No. Exacerbation last year	2.8 ± 0.78	3 ± 0.82	2.9 ± 0.51	3.3 ± 0.81	3 ± 1.4	0.62
No. hospital admission last 2 y	1.2 ± 0.72	1.3 ± 0.85	1.3 ± 0.75	1.3 ± 0.52	1.5 ± 0.71	0.98

*P-value < 0.05

The table illustrated that there was a statistical significant higher level of both Modified Reiff score, and number of CT lobes among cases infected with staph with p-value 0.03, and 0.01 respectively. in addition, there was a statistical significant high percentage of mild Modified Reiff score among cases infected with influenza and moderate degree among cases infected with Klebsiella, but sever degree was found among cases infected with pseudomonas with p-value 0.003. (Table 9).

**Table 9:** Comparisons of types of growth with radiology severity among cases

Variables	Influenza	pseudomonas	Klebsiella	Staph	Mixed	*p*-value
	No.(%)	No.(%)	No.(%)	No.(%)	No.(%)	
	Mean ± SD	Mean ± SD	Mean ± SD	Mean ± SD	Mean ± SD	
Modified Reiff score	7.7 ± 2.9	10.4 ± 4.4	8.9 ± 1.9	11.8 ± 3.9	10 ± 1.4	0.03*
No.CT lobes	2.7 ± 0.95	3.6 ± 1.1	3 ± 0.85	4.2 ± 0.98	3 ± 0	0.01*
Modified Reiff score severity						
Mild	**13(54.2%)**	3(18.8%)	1(8.3%)	0(0%)	0(0%)	0.003*
Moderate	9(37.5%)	9(56.3%)	**11(91.7%)**	3(50%)	2(100%)	
Severe	2(8.3%)	4(25%)	0(0%)	**3(50%)**	0(0%)	

*P-value < 0.05

The post Hoc tests illustrated a statistically significant difference in Modified Reiff score, and number of CT lobes between H. influenzas and staph infection with higher mean among staph infection *p* = 0.01, and 0.03 respectively (Table 10).

**Table 10:** Post Hoc test for clinical measurements in different types of growth

Pairs	O2 saturation	FEV1 score	FACED score	BSI score	Cough morning	Cough night	Modified Reiff score	No. CT lobes
H.Influenzas vs. pseudomonas	0.18	1	1	1	1	1	0.15	0.85
H.Influenzas vs. Klebsiella	1	0.08	1	1	1	0.48	1	1
H.Influenzas vs. staph	0.41	0.49	1	1	1	0.99	0.03*	0.01*
H.Influenzas vs. mixed	0.53	1	1	1	1	1	1	1
pseudomonas vs. Klebsiella	1	1	1	1	1	1	1	1
pseudomonas vs. staph	1	1	1	1	1	1	1	1
pseudomonas vs. mixed	1	1	1	1	1	1	0.85	1
Klebsiella vs. staph	1	1	0.94	0.73	1	1	1	0.19
Klebsiella vs. mixed	**1**	**1**	**1**	**1**	**1**	**1**	**1**	**1**
staph vs. mixed	**1**	**1**	**1**	**1**	**1**	**1**	**1**	**1**

*P-value < 0.05

The post Hoc tests illustrated a statistically significant difference in Modified Reiff score between influenzas, Klebsiella and staph infection with higher percentage of severe degree in staph infection, then moderate degree among Klebsiella followed by mild degree in H.influenza infection *p* = 0.008, 0.01and 0.02 (Table 11).

**Table 11:** Post Hoc test for clinical Levels and degrees in different types of growth

Pairs	O2 saturation	FEV1 score	FACED score	BSI score	Modified Reiff score
H.Influenzas vs. pseudomonas	0.26	0.85	0.33	0.99	0.06
H.Influenzas vs. Klebsiella	0.61	0.06	0.98	1	0.008*
H.Influenzas vs. staph	0.11	0.32	0.12	0.36	0.01*
H.Influenzas vs. mixed	0.13	0.56	1	1	0.23
pseudomonas vs. Klebsiella	0.51	0.21	1	1	0.09
pseudomonas vs. staph	0.64	0.42	0.48	0.48	0.37
pseudomonas vs. mixed	0.54	0.57	1	1	0.48
Klebsiella vs. staph	0.32	0.32	0.33	0.33	0.02*
Klebsiella vs. mixed	0.23	0.23	-----	---	1
staph vs. mixed	0.80	0.80	1	1	0.46

*P-value < 0.05

## Discussion

Bronchiectasis is a pulmonary disease characterized by permanently dilated bronchi, visible on lung imaging, resulting in mucus impaction and inflammation of airways. It has many clinical characters, e.g. cough, purulent sputum and frequent infective exacerbation [12].

Classification of disease severity can help in treatment and follow-up. The most widely utilized scores to assess severity are the BSI (Bronchiectasis Severity Index) and FACED scores [6].

Conventionally, forced expiratory volume in 1s second (FEV1) used to represent disease severity by spirometry. The modified Reiff score has also used as marker of disease severity [7].

Obtaining a lower airway secretion specimen should be done in all bronchiectasis patients for routine bacteriological culture. It helps to choose appropriate antibiotics for exacerbations and chronic suppressive therapy. Previous sputum bacteriology is useful to decide type of antibiotic to use [5].

BAL samples the lower airways directly, reducing contamination with upper airway flora, more sensitive for organisms such as Pseudomonas aeruginosa, atypical mycobacteria, and Pneumocystis jirovecii, and can be used as therapeutic and diagnostic procedure.

The aim of this study is to detect the bacteriology of bronchiectasis patients and relation to disease severity. 60 patients were investigated at chest department of Fayoum university hospital who were admitted by exacerbation. BAL culture and sensitivity was done. Disease severity was assessed clinically by cough score, mMRC dyspnea score and oxygen saturation. No of lobes affected in CT chest and modified rieff score were done. Spirometry and classification of severity by FEV1, and finally FACED and BSI scores were calculated.

The mean age among study group was (45.9 ± 16.1) years old and mean BMI was (24.5 ± 5.9) with 40% were males versus 60% were females. 23.3% were smoker, 10% had diabetes mellitus, and same for hypertension. 21.7% of them were overweight, and 25% were obese.

Forced expiratory volume in 1s (FEV1) reflects degree of air way obstruction. In our study, 4 cases have mild, 18 have moderate, 21 have severe, 17 have very severe obstruction.

FACED score effectively predict mortality but it does not include the exacerbations number as a separate item [13]. I this study, 57 cases have mild, one has moderate and 2 have severe scores.

FACED and BSI scores predicted long-term mortality and showed similar results regarding severity and prognosis, but BSI showed annual risk and outcomes of hospitalization. In our study, 57 cases have mild BSI and 3 have moderate score.

There are many radiological score systems offering a way to monitor disease status and progression radiologically, modified Reiff score is a scoring system that describe the extent and severity. It assesses the number of lobes affected and the degree of bronchial dilatation, since then it is frequently used in studies and recommended for its simplicity [14]. In our study 28.3% showed mild, 56.7% showed moderate and 15% showed severe level of Modified Reiff score severity with a mean of (9.1 ± 3.5), and mean CT lobes affected (3.15 ± 1.05).

Regarding O2 saturation, 26 cases have normal saturation, 24 have mild hypoxia, 10 have severe hypoxia.

Regarding the clinical symptom the mean cough score at morning is 2.28 ± 0.61, at night is 2.62 ± 0.58 and mMRC dyspnea score is 3.4 ± 0.62.

In our study, BAL culure and sensitivity isolation of H.Influenza represents 40%, Pseudomonas represents 26.7%, Klebsiella represents 20%, Staph aureus represents 10% and Pseudomonas& Klebsiella represent 3.3%.

The strongest predictor of future exacerbations is the previous history of exacerbations [15]. Patients with ⩾3 exacerbations per year were at increased risk of mortality. The rate of hospitalization in bronchiectasis reflects bronchiectasis severity and are associated with declining lung function, increased risk of mortality and cost implications for health system [16]. In this study the mean number of exacerbation was (2.9 ± 0.75) and mean number of hospitalization times last 2 years was (1.3 ± 0.73).

The bacteria commonly isolated from airways of bronchiectasis patients are Haemophilus influenzae, Pseudomonas aeruginosa, Streptococcus pneumoniae, Moraxella catarrhalis, and Staph aureus [4]. Gram-negative non-fermenting bacteria such as Klebsiella, Achromobacter (Alcaligenes) xylosoxidans and Stenotrophomonas maltophilia [17].

In UK studies, the most frequent pathogen detected are Haemophilus influenzae, followed by P. aeruginosa, Moraxella catarrhalis, Streptococcus pneumoniae, Staphylococcus aureus and enterobacteriaceae [5].

In comparison with Miao XY et al., study the distributions of main bacterial strains were 29% for H. influenzae, 28% for P. aeruginosa, 11% for S. pneumoniae, 12% for S. aureus, and 8% for M. catarrhails [1].

In Murray M et al., study isolation rates of P. aeruginosa were 48.1%, and Haemophilus influenza is 40.7% and the isolation rates of other pathogenic bacteria were only 3% [18]. This difference likely due to Geographic differences and Sampling method (BAL vs. sputum).

In Rogers GB et al., study Haemophilus influenza represents 33.4%, followed by 23.5% Pseudomonas, 9.5% Prevotella, 8.2% Veillonella, 5.6% Streptococcus, 2% Pasteurella, 1.9% Neisseria, 1.6% Porphyromonas, and 1.5% Moraxella [19].

In our study we compare between BAL isolated organism and different disease severity assessment tools (oxygen saturation, spirometry, FACED score, BSI score, cough score mMRC score, modified reiff score, number of exacerbations and hospitalization).

P. aeruginosa is a main bronchiectasis pathogen with poorer outcome and higher mortality, number of exacerbations, worse quality of life, and deteriorated pulmonary function and radiographic findings than other pathogens [20].

In our study, the mean number of exacerbation was (2.9 ± 0.75) and mean number of hospitalization times last 2 years was (1.3 ± 0.73) with 58.3% of cases show bacterial growth higher percentage was for pseudomonas 40% for H.influenza infection, followed by 26.7% for infection with Pseudomonas. (Table 7).

In our study, there was a statistically significant lower mean of oxygen saturation level among cases infected with both pseudomonas and Klebsiella with p-value 0.04. On the other side, there was no statistically significant difference with p-value > 0.05 as regards other disease clinical severity variables. There was a statistically significant higher level of both Modified Reiff score, and number of CT lobes affected among cases infected with staph aureus with p-value 0.03, and 0.01 respectively. Also, there was a statistical significant high percentage of mild Modified Reiff score among cases infected with H. influenza and moderate degree among cases infected with Klebsiella, but severe degree was found among cases infected with pseudomonas with p-value 0.003.

In comparison with Rogers GB et al., study Patients with P. aeruginosa showed more lower FEV1 and had a greater number of exacerbations in the last 1 year than other organisms but no significant differences observed in clinical measures with comparison to H. influenzae–dominated patients [19].

In Byun MK et al., study there was no differences in lung microbial composition in patients with exacerbation compared to stable bronchiectasis [21].

Lee SH et al., study studied the sputum microbiome in bronchiectasis patients with comparison to disease severity assessed by FACED score. Haemophilus was the most common especially in the mild bronchiectasis group, while that of Pseudomonas was significantly predominant in the moderate and severe bronchiectasis groups [22]. 

## Conclusion

In our study H. influenzae consider as a major pathogen isolated by BAL culture in patients with bronchiectasis exacerbation admitted at chest department of Fayoum University Hospital, followed by P. aeruginosa, then klebsiella then S. aureus.

Cases infected with P. aeruginosa and klebsiella have the worst oxygen saturation. The highest modified Rieff score was in P. aeruginosa than other isolated organisms.

### Limitation

Small sample size, especially for pathogen subgroups.

Use of culture-based methods only.

Lack of longitudinal follow-up.

## Data Availability

The datasets used and/or analyzed during the current study are available from the corresponding author on reasonable request.

## Abbreviations

mMRC: Modified medical research council
BSI: Bronchiectasis severity index
FEV1: Forced expiratory volume in 1st second
HRCT: High resolution computed tomography
MRSA: Methicillin resistant staph aureus
CBC: Complete blood count
CRP: C reactive protein
ESR: Erythrocyte sedimentation rate
BMI: Body mass index
DM: Diabetes mellitus
HTN: Hypertension
RA: Rheumatoid arthritis
COPD: Chronic obstructive pulmonary disease
BAL: Broncho alveolar lavage
